# Implementation of balint group for a team who care patients with head and neck cancer in a service in Brazil: A proposal post qualitative research

**DOI:** 10.1192/j.eurpsy.2021.1723

**Published:** 2021-08-13

**Authors:** E. Turato, A.C. Bispo, J.R. Rodrigues, C. Santos, C.S. Lima

**Affiliations:** 1 Medical Psychology And Psychiatry, University of Campinas, Campinas, Brazil; 2 Lpcq - Laboratory Of Clinical-qualitative Research, University of Campinas, Campinas, Brazil; 3 Medical Psychology And Psychiatry, Unicamp, Campinas, Brazil

**Keywords:** oncoloy and psychology, medical psychology, Medical Education, Balint groups

## Abstract

**Introduction:**

The Balint group emerged at the Tavistock Clinic in London in the early 1950s. Its creator was a doctor and psychoanalyst Michael Balint. It consisted of a group process, with meetings among general practitioners, in which non-conscious aspects of the professional-patient relationship were approached. We present how a proposal for implementation of a Balint Group has emerged, specifically for physicians and nurses who care for cancer patients. Is is a consequence of results obtained from a qualitative study conducted by a student of the professional master’s degree linked to a Clinical Oncology.

**Objectives:**

To present a technical product, as required in a Brazilian professional master’s degree, as a result of research that studied reports of doctors and nurses who deal with usual difficulties of handling patients with HNC.

**Methods:**

The group work is triggered by the report of a case brought by a participant, presenting a problem-situation in the management of his patient. The meeting leader seeks to understand the reactions reported by the presenter in the light of a psychodynamic approach.

**Results:**

Expected results: the holding of a Balint group, perhaps monthly, in charge of a colleague who has knowledge in applied psychoanalysis, will allow insights to the participants who will bring them conditions to perceive “neurotic elements” in the relationship with their patient.

**Conclusions:**

Final consideration: having accumulated decades of positive experience, Balint Groups must remain as an updated proposal for the work on emotional issues of professional teams, with emphasis on clinical services with the management of so-called “difficult patients”.

**Disclosure:**

No significant relationships.

